# β-nicotinamide mononucleotide (NMN) production in *Escherichia coli*

**DOI:** 10.1038/s41598-018-30792-0

**Published:** 2018-08-16

**Authors:** George Cătălin Marinescu, Roua-Gabriela Popescu, Gheorghe Stoian, Anca Dinischiotu

**Affiliations:** 1 Department of Biochemistry and Molecular Biology, University of Bucharest, Bucharest, 050095 Romania; 2 Independent Research Association, Bucharest, 012416 Romania

## Abstract

Diabetes is a chronic and progressive disease with continuously increasing prevalence, rising financial pressure on the worldwide healthcare systems. Recently, the insulin resistance, hallmark of type 2 diabetes, was cured in mice treated with NAD^+^ precursor β-nicotinamide mononucleotide (NMN), no toxic effects being reported. However, NMN has a high price tag, more cost effective production methods are needed. This study proposes a biotechnological NMN production method in *Escherichia coli*. We show that bicistronic expression of recombinant nicotinamide phosphoribosyl transferase (Nampt) and phosphoribosyl pyrophosphate (PRPP) synthetase in the presence of nicotinamide (NAM) and lactose may be a successful strategy for cost effective NMN production. Protein expression vectors carrying NAMPT gene from *Haemophilus ducreyi* and PRPP synthetase from *Bacillus amyloliquefaciens* with L135I mutation were transformed in *Escherichia coli* BL21(DE3)pLysS. NMN production reached a maximum of 15.42 mg per L of bacterial culture (or 17.26 mg per gram of protein) in these cells grown in PYA8 medium supplemented with 0.1% NAM and 1% lactose.

## Introduction

According to International Diabetes Federation, the number of adults with type 2 diabetes was 425 million in 2017, and it is expected to hit 649 million by 2045. On a global scale, 12% of the healthcare budget (or $727 billion) is being spent on treating diabetes. Recent studies linked insulin resistance, the hallmark of type 2 diabetes, to the decline of mitochondrial function and decreased NAD^+^ levels as well as NAD^+^/NADH ratio, also observed in aging^1^. NAD^+^ is present in all living organisms and is a well-known coenzyme in oxidation-reduction reactions^2–5^. NAD^+^-dependent protein deacetylases such as SIRT1 and SIRT6 serve as metabolic sensors, and regulate downstream pathways, which eventually restore mitochondrial function and insulin sensitivity^6–13^. This finding extends the research domain of NAD^+^ effects to other degenerative diseases associated with aging, such as: cardiovascular, cancer, arthritis, osteoporosis or Alzheimer’s diseases^14–16^.

The β-nicotinamide mononucleotide (NMN)^17^ is an intermediate in NAD^+^ biosynthesis produced from nicotinamide (NAM) and phosphoribosyl pyrophosphate (PRPP) by nicotinamide phosphoribosyl transferase enzyme (Nampt) (EC 2.4.2.12)^7,18–21^. Being well tolerated, with no reported side effects during long term administration in mice, and preventing age-associated physiological decline^22^, NMN proved to be effective in treating high fat diet-induced type 2 diabetes^23^, by reversing mitochondrial dysfunction associated with aging^24^, and rescuing the effect of age-associated decline in neural stem cells^25^.

NAM is usually converted to NMN by enzymes involved in the NAD^+^ salvage pathways, such as Nampt^11^. The action of this enzyme constitutes the main NAD^+^ anabolic activity in the cell^4^. The regulation of mammalian or microorganisms nucleotide metabolism and biosynthesis usually proceeds by consumption of PRPP^26^. PRPP results from ribose-5-phosphate via both oxidative and non-oxidative branches of pentose phosphate pathway^27–29^.

In bacteria, NAM is most often converted to nicotinic acid (NA) by nicotinamidase, which is integrated in Preiss-Handler pathway, this being also the case of *Escherichia coli*^30^. In mammals, nicotinamidase activity was not reported; NAM is converted to NMN by one of the NAM phosphoribosyl transferase enzymes instead^4^. Although most of bacteria lack NAM phosphoribosyl transferase, the enzyme is expressed in *Haemophilus ducreyi*^31,32^ and *Shewanella oneidensis*^33,34^.

Simple and metabolically versatile organisms, such as *Escherichia coli*, are often used in biotechnology for controlled expression of proteins with desired enzymatic activity. The DNA coding for such enzymes is often introduced in bacteria by expression vectors such as pET system from Novagen^35–37^. The pET vectors are transformed in bacteria expressing T7 RNA polymerase, such as *E*. *coli* BL21(DE3). The enzymes expression control is achieved using *lac* promoter, which turns on the expression only in the presence of lactose (also metabolized as a carbon source) or its synthetic structural analogue, Isopropyl-1-Thio-β-D-galactopyranoside (IPTG) (not metabolized, with constant concentration during growing process)^38^. As bacterial metabolism is versatile, the variation of carbon source^39^ and concentration of medium supplemented enzyme substrates are key factors to take into consideration in a biotechnological process.

Aiming to address the current high price problem of NMN, in our previous work we proposed a purification method from bacterial cells^40^. For further process optimization, as we were able to find only one yeast production method published^41^, this study proposes a simple and cost effective NMN biotechnological production method in *Escherichia coli*.

## Results

### Bacterial transformation

The multiple amino acid sequences alignment generated for putative NadV from *Haemophilus ducreyi*, NadV, *Shewanella oneidensis* MR-1 and Nampt from *Mus musculus*, shows high similarity (Fig. S1), making all these enzymes candidates for a biotechnological process.

Transformed *E*. *coli* cells with nadV (NAMPT) genes carried by pET28a(+) plasmids formed colonies on the LB Agar plates supplemented with kanamycin. No colonies were detected on plates inoculated with untransformed bacterial cells. Agarose gel electrophoresis of plasmid DNA isolated from these colonies confirmed the presence of Nampt-PET28a, pET28a-soNadV or pET28a-hdNadV plasmids in *E*. *coli* DH5α (Supplementary Fig. S2A, lane 2, 3, 4). The DNA bands matched the bands from *E*. *coli* BL21(DE3)pLysS cells (Supplementary Fig. S2A, lane 6, 7, 8), which were transformed with corresponding plasmids extracted directly from *E*. *coli* DH5α. In untransformed *E*. *coli* BL21(DE3)pLysS cells, used as negative control, the presence of the pLysS plasmid was confirmed by electrophoresis, a band matching the known length of 4886 bp (Supplementary Fig. S2A, lane 5) was shown on agarose gel.

### The growth curve of the transformed bacteria

#### Determination of tolerated NAM concentration

From a biotechnological perspective, it was questioned whether nadV (NAMPT) gene products from pET28a(+) plasmids or NAM used as precursor for NMN were toxic for bacterial cells, preventing the culture to reach high densities during bio-synthetic processes.

**Figure 1:**
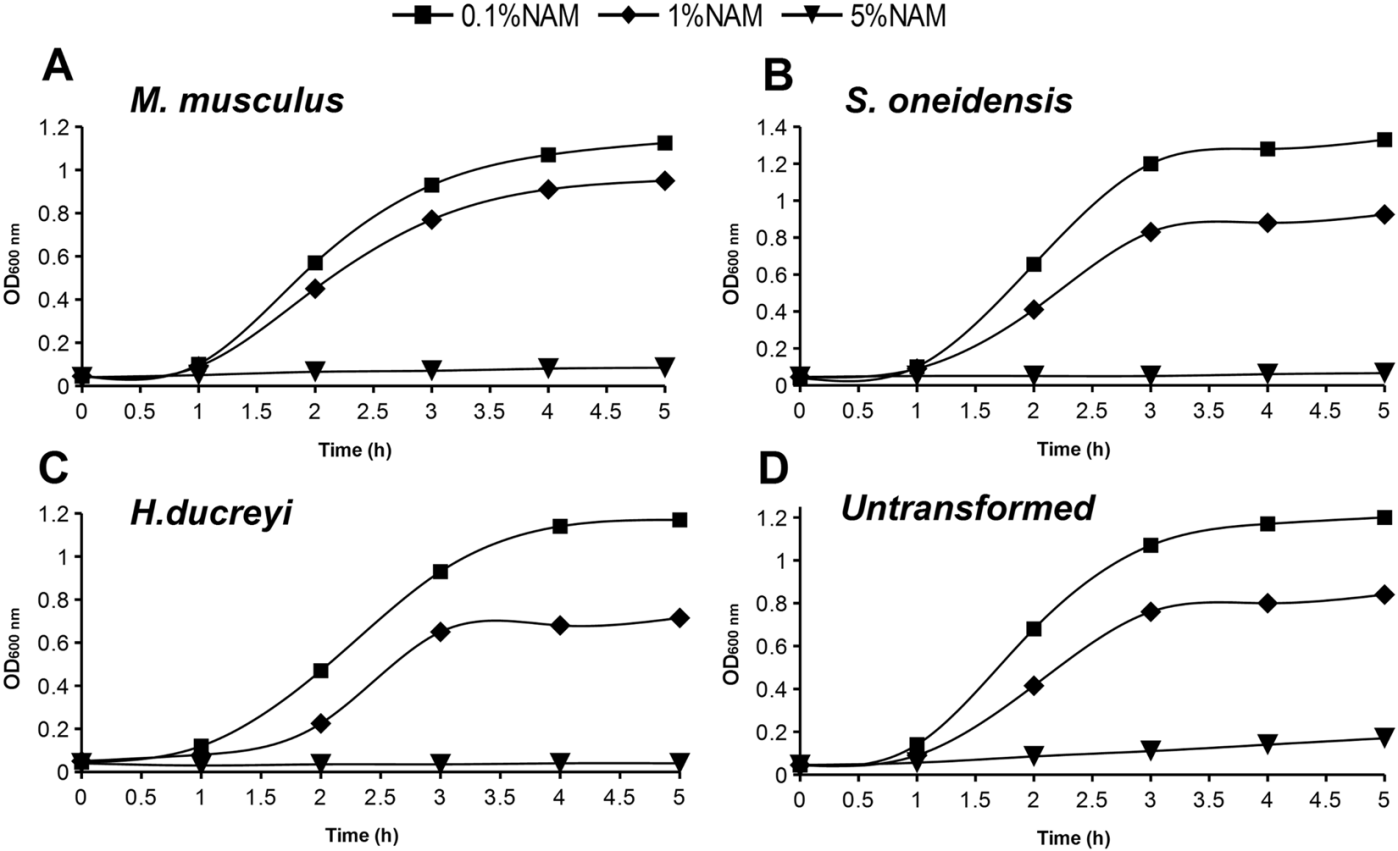
The growth curve (OD600) of transformed *Escherichia coli* BL21(DE3)pLysS with pET-28a(+) vector carrying nadV (NAMPT) gene (**A**–**C**) respectively untransformed culture (**D**) in LB medium supplemented with 0.1%, 1% and 5% NAM.

Resulted growth curves of transformed and untransformed strains in medium supplemented with different NAM concentration (0.1%, 1%, 5%) are shown in Supplementary Fig. S3. LB broth supplemented with 0.1 and 1% NAM respectively, did not inhibit growth completely. However, the growth rates dropped considerably when 5% NAM was used (Supplementary Fig. S3C, Fig. 1). Using the lowest NAM concentration (0.1%), the highest optical density was reached in bacterial cells transformed with pET28a-soNadV (OD_600nm_ > 1.3), 1% NAM being well tolerated (OD_600nm_ reached 1) in shake flasks culture (Supplementary Fig. S3A).

### Expression of recombinant enzymes NadV (Nampt)

Three hours after inoculum, samples from shake flasks cultures of bacterial cells expressing nadV (NAMPT) genes were collected to evaluate recombinant protein expression by SDS-PAGE (Supplementary Fig. S2D, lane 2–5). In the remaining cultures, IPTG was added to induce the expression of recombinant proteins NadV (Nampt). Three hours later, samples were harvested and subjected to SDS-PAGE (Supplementary Fig. S2D, lane 6–9), in order to confirm the presence of the recombinant NAMPT proteins from *Mus musculus* (Supplementary Fig. S2D, lane 6), nadV from *Shewanella oneidensis* (Supplementary Fig. S2D, lane 7), nadV from *Haemophilus ducreyi* (Supplementary Fig. S2D, lane 8) corresponding to the known size of 55 kDa.

### Determination of optimal OD_600 nm_ for the induction of recombinant enzymes expression

#### Determination of optimal OD_600 nm_ for culture harvest

Cultures of untransformed (nadV−) and transformed (nadV+) with pET28a-hdNadV vector in *E*. *coli* BL21(DE3)pLysS strain were inoculated simultaneously. Each hour, during a four-hour period, IPTG was added for induction of nadV (NAMPT) expression. All cultures were harvested at OD_600nm_ = 0.85 and subjected to NMN quantification. Induction at OD_600nm_ = 0.46 produced the highest NMN concentration (Supplementary Fig. S4). A higher optical density of culture at the time of IPTG induction led to lower maximum intracellular NMN concentration. The most favorable time for harvest was three hours after the induction of nadV expression at OD_600nm_ = 0.73.

#### NMN yield of each recombinant enzyme

**Figure 2:**
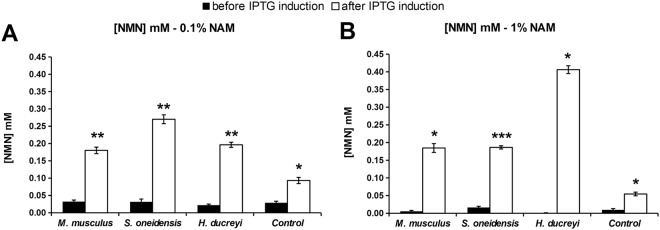
Intracellular NMN concentrations in transformed *Escherichia coli* BL21(DE3)pLysS culture with recombinant nadV (NAMPT) genes versus untransformed (control) grown in LB medium supplemented with 0.1% NAM (**A**) and 1% NAM (**B**). *P ≤ 0.05; **P ≤ 0.01; ***P ≤ 0.001.

As observed in Supplementary Fig. S3C, 0.1% and 1% NAM concentrations were well tolerated by bacteria, therefore NMN production was determined only for cultures in a medium supplemented with these two NAM concentrations. In all shake flask cultures, transformed cells with any of the three plasmids produced significantly higher intracellular NMN, comparative to untransformed cells. The nadV gene from *Haemophilus ducreyi* led to the highest increase in intracellular NMN concentration, by 7.44 times, compared to untransformed bacteria. (Fig. 2).

#### Co-expression of PRPP synthetase and Nampt

**Figure 3:**
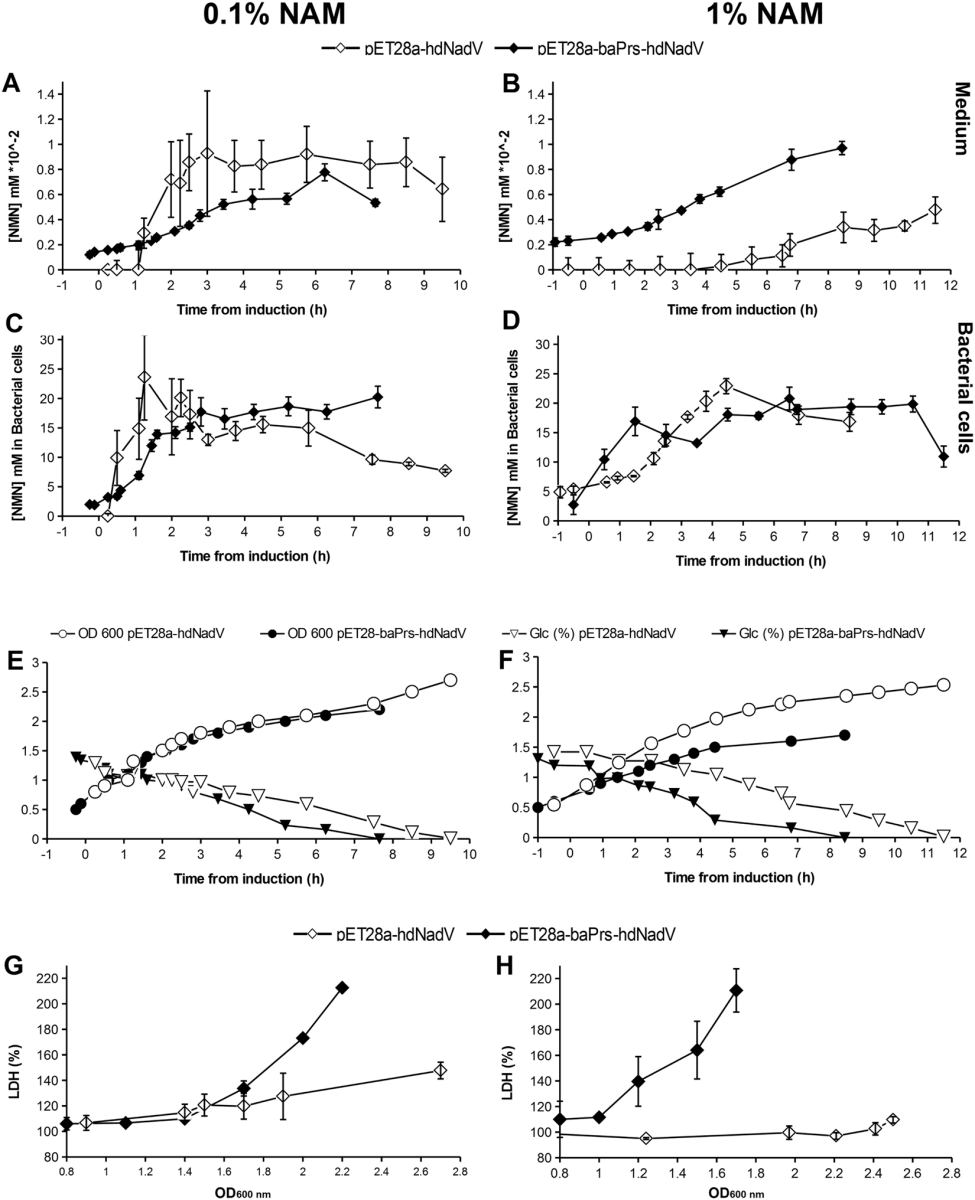
NMN production kinetics, glucose uptake, NAM concentration effect (0.1% vs 1%) in LB growth medium supplemented with 1% glucose. *Escherichia coli* BL21(DE3)pLysS cultures with pET28a-hdNadV respective pET28a-baPrs-hdNadV grown in 500 mL bioreactor were sampled at shown time intervals relative to IPTG induction time for medium (**A**,**B**) and intracellular (**C**,**D**) NMN concentration assay. Culture density kinetics and glucose uptake during growing process (**E**,**F**) is shown. Cells mortality was evaluated by lactate dehydrogenase assay (LDH) from growth medium samples (**G**,**H**).

**Figure 4:**
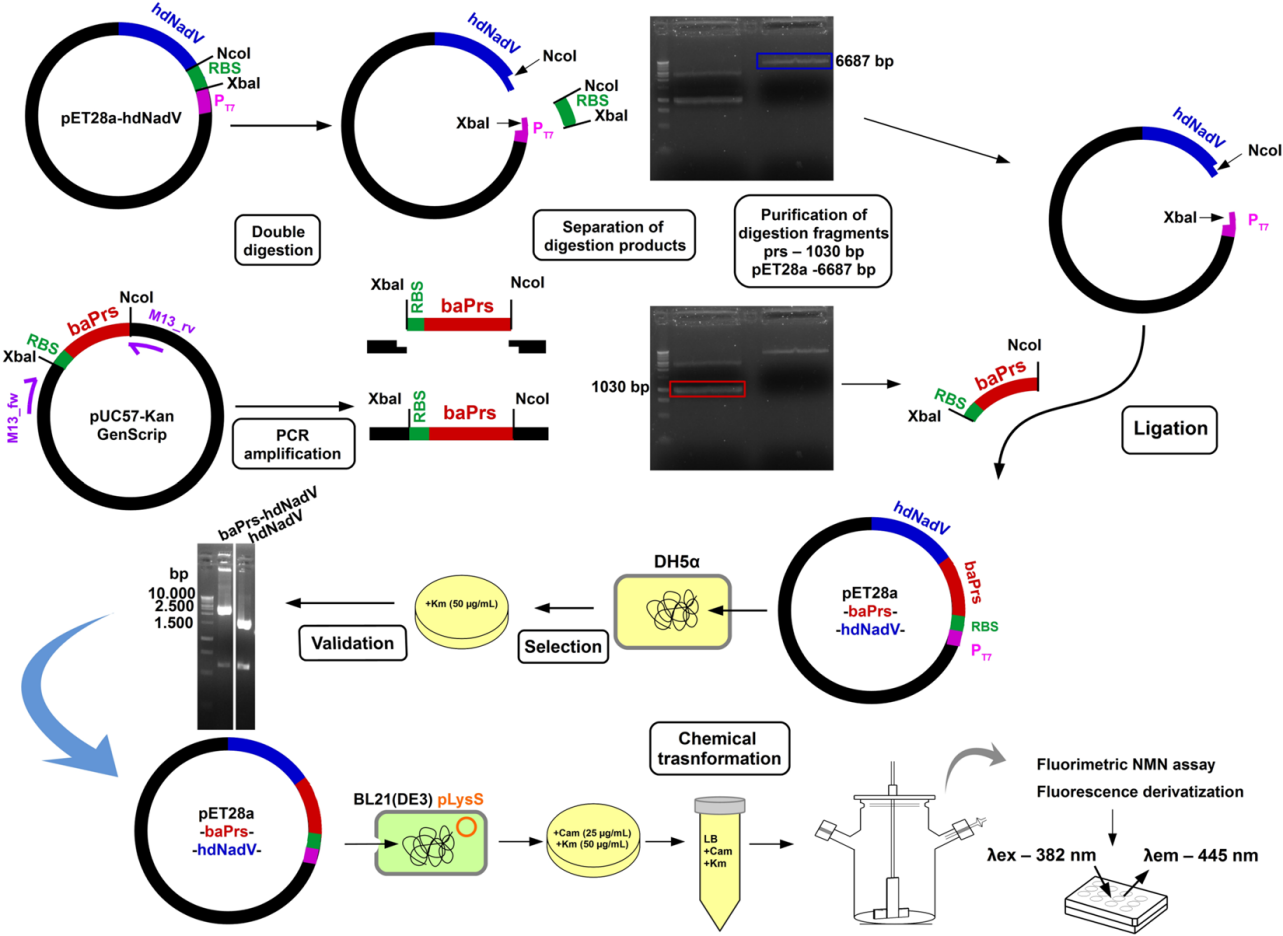
pET28a-baPrs-hdNadV bicistronic vector construction. Prs gene from *Bacillus amyloliquefaciens* with L135I mutation (ctc to ata) was cloned in pUC57-kan vector (GenScript synthesis). pET28a-hdNadV Vector and PCR amplification of prs fragment were digested (with NcoI and XbaI restriction enzymes) and the fragments corresponding to 1030 bp and 6687 bp were purified and ligated. The DNA construct was chemically transformed in *Escherichia coli* DH5α, verified by agarose gel electrophoresis and transformed in strain BL21(DE3)pLysS. After transformation, cells were grown into a 500 mL bench-top bioreactor system and the NMN yield was determined by derivatization followed by fluorimetric assay.

Besides NAM, the other substrate for the reaction catalyzed by NAMPT is PRPP. PRPP synthetase from *Bacillus amyloliquefaciens* with L135I mutation was formerly expressed in *E*. *coli* BL21(DE3)pLysS for biotechnological purposes. This mutation was previously demonstrated to eliminate the negative feedback allosteric inhibition of PRPP synthetase and led to high level of PRPP^29^. *E*. *coli* BL21(DE3)pLysS cells transformed with bicistronic vector, grown in LB broth supplemented with 1% glucose and NAM (0.1% respectively 1%), produced a maximum of 18.16 mg NMN per gram of protein (Fig. 3C,D). Bicistronic expression produced similar intracellular NMN concentration, but by 2.46 times higher than similar cells carrying the two genes on separate plasmids (Table 1). We investigated comparatively the effect of IPTG and lactose expression induction on NMN production by adding these compounds into similar cultures of LB media containing *E*. *coli* BL21(DE3)pLysS cells transformed with the bicistronic expression plasmid (pET28a-baPrs-hdNadV) constructed as shown in Fig. 4. When the two proteins were expressed individually (co transformed cells with both pET28a-hdNadV and pER15b-PRS135 plasmids) in lactose supplemented medium, the NMN production was lower (7.36 mg per gram of protein) than the culture in which the genes corresponding to Nampt and PRPP synthetase were cloned into the same vector (16.06 mg per gram of protein).

**Table 1:** NMN production in *E*. *coli* BL21(DE3)pLysS transformed cells.

Vector	Growth medium	NAM (%)	Glucose (%)	Inductor	[NMN] mM in bacterial cells	[NMN] mg per gram of protein	[NMN] mg per L of culture	OD 600
pET28a-hdNadV	LB	0.1	1	IPTG	23.57 ± 7.2	18.70 ± 5.73	10.89 ± 2.5	1.3–1.6
	LB	1	1	IPTG	20.79 ± 1.92	16.63 ± 1.52	14.33 ± 0.76	2.2–2.4
pET28a-baPrs-hdNadV	LB	0.1	1	IPTG	20.24 ± 1.84	16.06 ± 1.46	13.69 ± 1.17	2.1–2.2
	LB	0.1	—	Lactose	16.86 ± 3.27	13.37 ± 2.60	8.57 ± 1.56	1.6–1.8
	PYA8	0.1	—	Lactose	22.63 ± 2.14	17.26 ± 1.63	15.42 ± 1.47	2.3–2.6
	LB	1	1	IPTG	22.89 ± 1.28	18.16 ± 1.01	11.06 ± 0.63	1.4–1.6
pET28a-hdNadV and pET15b-PRS135 (co-transformed separate vectors)	LB	0.1	1	IPTG	9.28 ± 0.36	7.36 ± 0.28	6.95 ± 0.54	1.3–2.2

### Growth media optimization

#### Bioreactor scale growth and NMN production kinetics

Up scaled culture to 500 mL bench-top bioreactor in LB medium supplemented with 1% glucose and 1% NAM increased NMN production in *E*. *coli* BL21(DE3)pLysS pET28a-hdNadV cells 29 times compared to shake flasks cultures (Fig. 2) without glucose addition, to a maximum of 20.79 mM NMN (or 16.63 mg NMN per gram of protein). The supplementation of the growth medium with 0.1% NAM and 1% glucose increased NMN production by 32.7 folds to a final value of 23.57 mM (or 18.7 mg per gram of protein) in bacterial cells (Fig. 3C,D). In order to evaluate if NMN was exported into the medium by living bacteria or leaked from dead cells, for both NAM concentrations and plasmids, the NMN concentration in culture media (extracellular) was measured (Fig. 3A,B) in IPTG induced cultures. In all these conditions, NMN concentration in media was below 1 × 10^−2^ mM. The level of lactate dehydrogenase (LDH) released in the growth media (Fig. 3G,H) suggested higher cells viability in 0.1% NAM supplemented cultures, while higher cell mortality was observed in cultures carrying the bicistronic expression plasmid (Fig. 3G,H). Bacterial cells carrying any of the two plasmids have shown similar glucose uptake pattern, consuming around 1 g/L glucose per hour Aeration, glucose supplementation, pH and temperature control of the bioreactor grown cultures were monitored in order to obtain a higher cells density compared to the cultures typically obtained in shake flasks. While cultures carrying only nadV gene revealed similar growth patterns in both NAM concentrations, those carrying the bicistronic plasmid reached a lower maximum cell density in 1% NAM (Fig. 3E,F).

**Figure 5:**
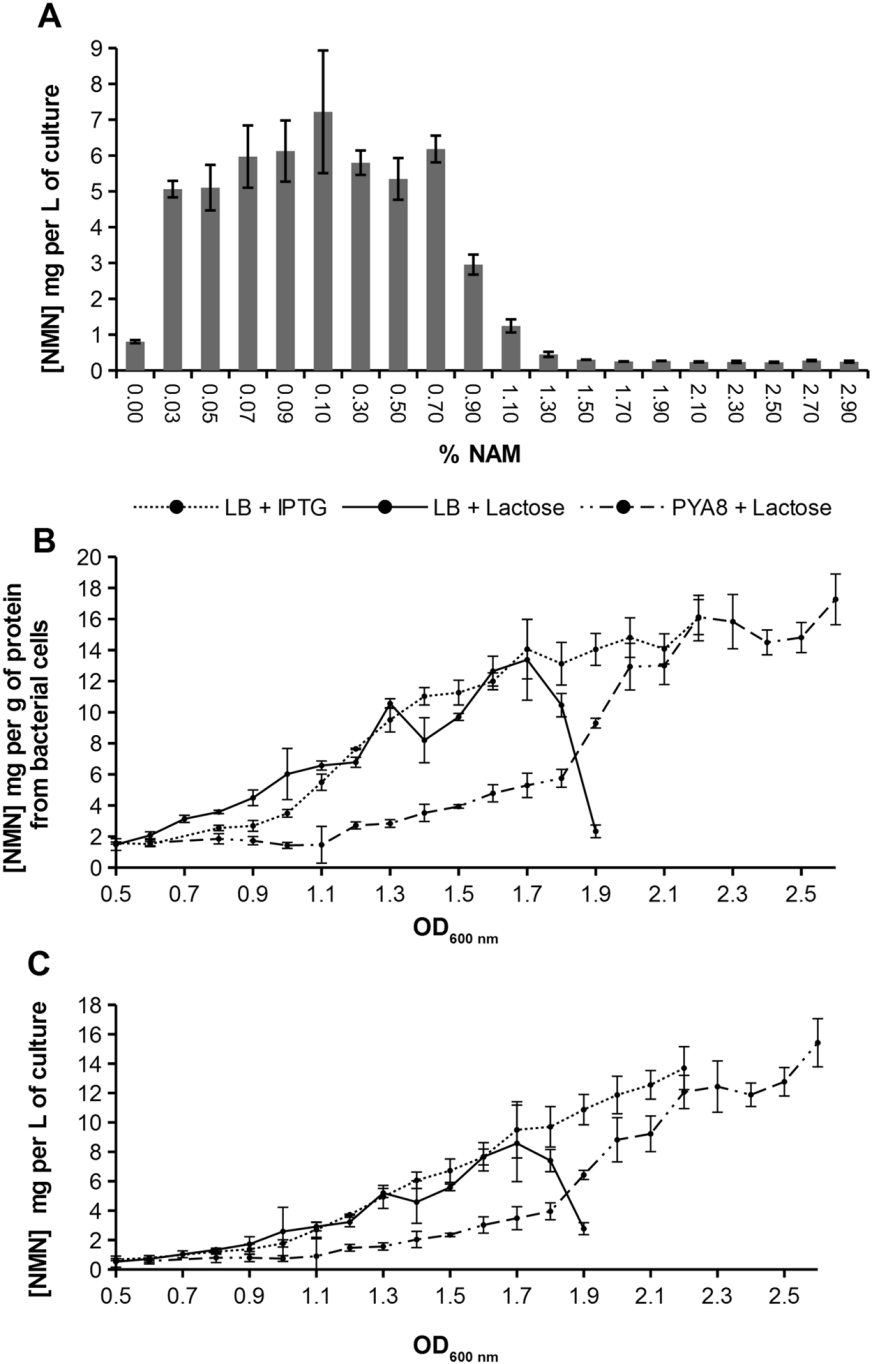
NMN yield in *E*. *coli* BL21(DE3) pLysS pET28a-baPrs-hdNadV grown in PYA8 + 1% Lactose + specified NAM concentrations for 12 hours (**A**); NMN production kinetics in *E*. *coli* BL21(DE3) pLysS pET28a-baPrs-hdNadV in LB medium supplemented with IPTG, LB medium + Lactose, respective PYA8 + Lactose. 0.1% NAM was added to each of these growing media. Data reported as mg NMN per g of protein from bacterial cells (**B**) and mg NMN per L of bacterial culture (**C**).

Varying NAM substrate concentration between 0 and 2.9%, resulted in a NMN yield peak corresponding to the culture grown in 0.1% NAM supplemented medium (Fig. 5A).

There were no major changes in NMN yield between bacterial cultures induced with 1% lactose versus 1 mM IPTG (Fig. 5) until the culture densities (OD_600nm_) reached the value of 1.7, confirming that lactose is a good but less expensive IPTG substitute. The sharp drop of NMN concentration after OD_600nm_ = 1.7 is probably due to the depletion of lactose which is metabolized by bacteria. Higher density cultures were obtained replacing LB with PYA8 medium as it was previously shown by Leone *et al*.^39^. NMN concentration in PYA8 + Lactose grown cells was significantly lower than that of the same cells grown in LB + Lactose or LB + IPTG at lower culture densities, but at culture densities over OD_600nm_ = 1.9, PYA8 medium was the most productive, NMN production reaching a maximum of 15.42 g/L of culture (or 17.26 mg NMN per gram of protein) at OD_600nm_ = 2.6 (Fig. 5, Table 1).

## Discussion

Taking into consideration the potential implications of NMN in the treatment of type II diabetes, in order to address the high cost problem of this compound, we propose a biotechnological NMN production method in *E*. *coli*, widely used in biotechnology. We were unable to find any production method in the scientific literature for this compound, except one patent showing some yeast production data. We followed the straight forward approach i.e. exploiting the natural NAD^+^ salvage pathway which converts NAM to NMN by a NAMPT enzyme. But *E*. *coli* lacks NAMPT enzyme. It has an alternative salvage pathway for NAD^+^ biosynthesis that is not interesting from a biotechnological point of view, as the involved intermediary metabolites are more expensive than NAM^30^. According to the tree of life^33^, most of the microorganisms expressing Nampt were either pathogenic or not previously used in biotechnology. As no information was found about the activity of these enzymes in general and about their expression in *E*. *coli*, we have decided to select three of them and comparatively evaluate their productivity. The amino acids sequences comparison of nadV genes from *Haemophilus ducreyi*, *Shewanella oneidensis* and NAMPT gene from *Mus musculus* showed that they are highly conserved (Supplementary Fig. S1), but we couldn’t make any assumption about their comparative productivity. *Haemophilus ducreyi*, *Shewanella oneidensis* were selected for their proximity to *E coli* in the tree of life. Previous attempt to transform pNAD1 plasmid into *E coli* DH5α strain was unsuccessful^42^, probably due to either codon unavailability or other elements incompatible with *E coli* strain present in plasmid pNAD1. Therefore we took from the database (UniProt, accession no. NP_957670.1) the nadV amino acids sequence carried on a pNAD1 plasmid, and optimized it in Genome Compiler software v. 2.2.55 (Genome Compiler Corporation 2015) for expression in *E*. *coli*. The DNA sequences of nadV genes of these two organisms obtained by synthesis (GenScript) were cloned in pET-28a+ vector while NAMPT from *Mus musculus* was conveniently available on pET-28a(+) *E*. *coli* expression vector at Addgene.

All these plasmids were transformed for long term storage in *E*. *coli* DH5α and for protein expression in BL21(DE3)pLysS. Successful transformation was confirmed by agarose gel electrophoresis, showing bands corresponding to 6704 bp–6788 bp in transformed bacterial cells (Supplementary Fig. S2A). The migration of pET28a-hdNadV and pET28a-soNadV plasmids in three bands was associated with the different conformations of the plasmid DNA, which are known to run at different speeds in agarose gel during electrophoresis^43^. The intensity of the band corresponding to the supercoiled conformation in comparison with the linear form is known to depend on the numbers of manipulations that are incurred by plasmid during the purification process. The nadV/NAMPT recombinant enzymes were confirmed by SDS-PAGE (Supplementary Fig. S2D).

No information about the tolerated concentration of NAM in the culture media for NMN production by transformed/untransformed bacteria was found in the scientific literature. Therefore we evaluated it in shake flasks cultures and found that a concentration of 5% NAM prevented bacterial growth, 1% had mild effect on cells growth, while 0.1% concentration was well tolerated. All the NAM supplemented cultures show doubling times longer than wild type *E*. *coli*. The observed growth slowing effect is NAM concentration dependent, (Supplementary Fig. S3, Fig. 1). The same pattern was observed for all three plasmids and also in untransformed bacteria, suggesting that it is the direct effect of NAM and not of plasmids or recombinant proteins. We decided to go further in evaluating the most productive nadV/0NAMPT enzyme in both 0.1% and 1% NAM supplemented media. All the transformed bacteria produced significantly higher NMN intracellular concentration than the control ones. The gene from *Haemophilus ducreyi* (Fig. 2) was the most productive, therefore it was selected for further evaluation of the optimal OD _600nm_ for the induction of protein expression and optimal OD_600nm_ for culture harvest.

NadV*/*NAMPT genes, delivered by pET-28a(+) vectors, were expressed under the control of a T7 lac promoter. *E coli* BL21(DE3)pLysS strain carried genomic DNA coding sequence for T7 RNA polymerase necessary for pET vectors transcription. Both T7 RNA polymerase and NadV/NAMPT enzymes transcription were controlled by lac promoter, which initiated transcription only in the presence of lactose or its non-metabolizable analog, IPTG^38^. The basal expression of recombinant proteins was reduced by a protease, product of pLysS plasmid. No basal protein expression was observed on SDS-PAGE (Supplementary Fig. S2D). The optimum cell culture density for inducing proteins expression was OD_600nm_ = 0.46. NMN production reached a peak at OD_600nm_ = 0.73 after 2 hours. Then the decline started, and higher cell densities resulted in lower NMN production (Supplementary Fig. S4).

Nampt catalyzes NMN biosynthesis transferring a phosphoribosyl group from phophoribosyl pyrophosphate (PRPP) to nicotinamide (NAM)^21^. NAM was provided to cells by supplementing directly the medium. PRPP is synthesized by phosphoribosyl pyrophosphate synthetase (PRPP synthetase, EC 2.7.6.1). Zakataeva *et al*.^29^ have shown that the expression of PRPP synthetase from *Bacillus amyloliquefaciens* strain IAM1523 with L135I mutation within pET15b(+) vector in *E*. *coli* led to PRPP overproduction. Suspecting that a NMN decline in higher density cultures might be caused by PRPP depletion, we co-transformed *E coli* BL21(DE3)pLysS pET28a-hdNadV with pET15b-PRS135 (a gift from Zakataeva N., Ajinomoto-Genetika Research Institute – AGRI, Moscow, Russia). This approach did not produce the desired results, NMN production dropped by 50% compared to pET28a-hdNadV only culture, probably due to additional DNA synthesis effort or lack of *E coli* codon optimization in prs L135I gene carried on pET-15b(+) (Table 1). Then we constructed a bicistronic vector inserting baPrs gene with L135I mutation and codon optimized obtained by synthesis (GenScript) into the existing pET28a-hdNadV plasmid as shown in Fig. 4.

Comparative NMN production kinetics of scaled up cultures in a 500 mL bioreactor with 1% glucose supplemented LB media shows that pET28a-hdNadV transformed cells reached a maximum NMN concentration 29 times higher compared to cultures where no glucose was added. Supplementing the medium with 0.1% NAM and 1% glucose, NMN production increased by 32.7 folds to a final value of 18.7 mg per gram of protein from bacterial cells (Fig. 3C,D). Probably, during the fermentation process, some cells were lysed and therefore, small amounts of NMN were detected in the growth medium (Fig. 3A,B). After the amino acids from LB medium were consumed, bacterial cells used around 1 g/L glucose per hour (Fig. 3E,F). The lactate dehydrogenase (LDH) release in the growing LB medium supplemented with 0.1% compared to 1% NAM (Fig. 3G,H) confirmed higher cells viability in 0.1% NAM media. Both monocistronic (pET28a-hdNadV) and bicistronic (pET28a-baPrs-hdNadV) transformed cells have shown almost identical growing patterns and glucose uptake in 0.1% NAM. A concentration of 1% NAM seemed to be, however, more toxic for the bicistronic plasmid carrying cells, both LDH and NMN concentration in growing media being higher (Fig. 3B,D).

From a biotechnological point of view, the production pattern of bicistronic plasmid is more favorable as NMN concentration is constantly rising, compared to the sharp maximum production peak in monocistronic transformed cells. It is difficult, if not impossible, to harvest exactly on the concentration peak. Therefore a continuously rising, predictable pattern, as the cells carrying the bicistronic vector show (Fig. 3C), is desirable, even though maximum concentration is slightly lower.

Although glucose cultures performed well, using IPTG for protein expression induction is not desirable in biotechnology because it is extremely expensive. Lactose may be used instead, but bacteria consume it as a carbon source (Fig. 5). In order to avoid a production drop when lactose is depleted, lactose should be continuously added during the growth process. To address this problem, we replaced LB with PYA8 medium which contains acetate, a more preferred carbon source than lactose. PYA8 is also a buffer, simplifying the process equipment, as pH compensation is not necessary anymore, it does not foam and is cheaper.

As both 0.1% and 1% NAM concentrations show acceptable NMN yields, from the product purification perspective, a lower NAM concentration is desirable, while higher NAM concentration may lead to higher yield. 0.03% NAM produced 5 times more NMN than medium without NAM, The NMN concentration was then rising up to the NAM concentration point of 0.1% and then started to drop. Starting with 1.3% NAM, bacterial growth was severely impaired and our results show that higher NAM concentrations are toxic for this strain (Fig. 5A).

The only previously published method for NMN production shows productivity values from 5 to “more than 50 mg per gram of protein” in yeast after filtration through a 10 kDa membrane^41^. Beside the unspecified maximum value, the 10 kDa membrane reduce substantially the protein content relative to which this concentration was reported, as higher molecular mass and insoluble proteins and complexes were filtered out. Therefore we consider our value of 17.26 mg NMN per gram of protein reported to whole protein content of the raw cells lysate the best reported so far.

The low cost of the growing media, simple and inexpensive process equipment required may lead to a price reduction of NMN to a level suitable for treating type 2 diabetes and aging related diseases in humans.

## Methods

### Bacterial strains

*E*. *coli* DH5α purchased from Life Technologies, genotype F− Φ80*lac*ZΔM15 Δ(*lac*ZYA-*arg*F) U169 *rec*A1 *end*A1 *hsd*R17 (rK−, mK+) *pho*A *sup*E44 λ− *thi*-1 *gyr*A96 *rel*A1 λ- was used for plasmids clonning and long term storage. *E*. *coli* BL21(DE3)pLysS purchased from Agilent, genotype B F^−^ *dcm ompT hsdS*(*rB*^−^*mB*^−^) *gal* λ(DE3) [pLysS Cam^r^] were used for protein expression.

### Protein expression vectors

Recombinant Nicotinamide phosphoribosyltransferase (Nampt) gene was cloned in pET-28a(+) expression vector from Novagen which permits protein expression under the control of T7*lac* promoter^35^. Three such plasmids, carrying expression DNA corresponding to each of the amino acid sequences compared in Fig. 1 were produced as follows: Nampt-PET28a vector containing Nampt gene from *Mus musculus* strain C57BL/6 J, a gift from dr. Cynthia Wolberger (Addgene plasmid # 25630), was cloned in *E*. *coli* DH5α and maintained (as a glycerol stock) at −80 °C. pET28a-hdNadV and pET28a-soNadV plasmids carrying the nucleotide sequence corresponding to nadV gene from *Haemophilus ducreyi* (strain: ATCC 27722), respectively *Shewanella oneidensis* (strain: MR-1) were constructed as follows: The gene sequences were selected from the GenBank database (Gene ID: 2716561 respectively 1169740), optimized for expression in *E*. *coli* (using Genome Compiler software v. 2.2.55 (Genome Compiler Corporation 2015), considering the frequency of codon usage and GC content, eliminating unwanted restriction sequences), synthesized *de novo* (GenScript) and ligated into pET-28a(+) vector which was first double digested with NcoI and XhoI restriction enzymes. The resulted plasmids were chemically transformed into competent *E*. *coli* DH5α. The transformed kanamycin (km) resistant bacteria were selected on agar plates. For pET28a-soNadV plasmid, for technical reasons a restriction site for NcoI enzyme (5′C^▼^CATGG3′) was added, assuming no effect on the enzyme activity.

pUC57-Kan-prs plasmid was generated by ligation of Prs gene sequence (Gene ID: HQ636460.1) with L135I mutation (CTC to ATA) synthesized by GenScript and ligated into NcoI/XbaI restriction sites of pUC57-Kan plasmid.

For the simultaneous expression of Nampt and PRPP synthetase a bicistronic vector was constructed by PCR amplifying the baPrs sequence (Q5 High-Fidelity 2X Master Mix, NEB) from pUC57-Kan plasmid with M13 forward and reverse primers, double digestion with XbaI and NcoI restriction enzymes (NEB) of PCR product and pET28a-hdNAdV plasmid, followed by the separation of the desired DNA fragments on 1.5% agarose gel electrophoresis^44^, purification from gel (using Wizard SV Gel and PCR Clean-Up System, Promega, Madison, USA) of desired bands (pET28a-hdNadV and baPrs) followed by ligation with T4 DNA ligase (NEB) (as shown in Fig. 4). Resulted bicistronic vector pET28a-baPrs-hdNadV was deposited at Addgene under the ID #91950.

The vectors presence was verified by 1.5% agarose gel electrophoresis and by PCR amplification with T7 forward and reverse primers (Supplementary Fig. S2). Cloning and transformation was performed into the expression strain *E*. *coli* BL21(DE3)pLysS in order to express the Nampt enzymes. pET28a-soNadV and pET28a-hdNadV vectors were deposited at Addgene under the IDs #83363 respectively #83362.

### Bacterial growth conditions

For plasmid cloning purposes, initial evaluation of recombinant protein expression, determination of tolerated NAM concentration in growing medium, optimization of protein induction and cell density, bacteria was grown in a shake flasks (250 rpm) incubator at 37 °C in Luria-Bertani (LB) medium^45^ supplemented with antibiotics for plasmid maintenance (kanamycin 50 μg/mL for expression vectors, 25 μg/mL chloramphenicol for pLysS plasmid). Bacterial growth kinetics was recorded as cell density, measured as 600 nm light scattering using Jasco 530 spectrophotometer.

Influence of glucose supplementation on growth kinetics and NMN production in *E*. *coli* BL21(DE3)pLysS with pET28a-hdNadV, pET15b-PRS135 (a gift from Zakataeva N., Ajinomoto-Genetika Research Institute – AGRI, Moscow, Russia) and pET28a-hdNadV, or pET28a-baPrs-hdNadV vectors was evaluated by inoculation of bacterial cells into a 500 mL benchtop bioreactor system. The cells were grown in LB medium supplemented with 0.1% or 1% NAM and 1% glucose and in PYA8 medium (1.61% Na_2_HPO_4_; 0.136% KH_2_PO_4_; 0.05% NaCl; 0.5% yeast extract; 1% CH_3_COONa). The growth conditions (37 °C, OD_600_, pH 7.0–7.2 and stirring motor speed at 150 rpm) were maintained bybioreactor system running open-source Arduino Software. Protein expression was induced by pumping Isopropyl-1-Thio-β-D-galactopyranoside (IPTG) to a final concentration of 1 mM when OD_600_ reached 0.7 or by auto-induction with 1% lactose. One milliliter from fermentation broth was harvested at every 0.1 units increase in optical density. The collected samples were centrifuged for 5 minutes at 2200 *g*. The supernatant and the sediment (resuspended in 1 mL water) were separately stored at −20 °C until the NMN yield and glucose concentration were determined.

For NAM substrate concentration optimisation, *E*. *coli* BL21(DE3)pLysS cells transformed with the plasmid pET28a-baPrs-hdNadV were grown in TPP 12 wells plates, in PYA8 medium supplemented with 1% lactose and 20 concentrations (3 wells for each concentration) of NAM (0, 0.03, 0.05, 0.07, 0.09, 0.1, 0.3, 0.5, 0.7, 0.9, 1.1, 1.3, 1.5, 1.7, 1.9, 2.1, 2.3, 2.5, 2.7, 2.9%), in a shaking incubator at 37 °C. After 12 hours, all cells were harvested and Fluorimetric NMN assay was performed.

### Bacterial cells viability evaluation

Lactate dehydrogenase (LDH) accumulation in medium during the fermentation process was determined using a commercially available kit (TOX-7, Sigma) and carried out according to the manufacture’s instructions.

### Transformation of bacteria and protein expression

Chemically competent *E*. *coli* cells were prepared by a protocol adapted from Seidman *et al*.^46^. Approximately 100 μL chemically competent cells were mixed with 1 μL plasmidial DNA resulted either from miniprep (Nampt-PET28a) or plasmids carrying synthesis DNA (pET28a-soNadV, pET28a-hdNadV or pUC57-Kan) (0.2 μg/µL). Tubes were placed for 30 minutes on ice, followed by heat shock for 45 seconds in a water bath preheated at 42 °C, then cooled on ice for 2 minutes. Then, a volume of 900 μL of LB medium was added and incubated for one hour at 37 °C under shaking conditions at 250 rpm. The cells were inoculated on agar Petri dishes with specific antibiotics for selection (10 μL/plate) and were left overnight at 37 °C. Next day, single colonies were selected from each plate and inoculated in LB medium containing the antibiotics for plasmids maintenance. After one day, plasmid DNA was extracted from 1 mL of overnight culture using “Fast-n-Easy Plasmid Mini Prep Kit” from Jena Bioscience. The presence of the cloned vectors was confirmed by 1% agarose gel electrophoresis. DNA concentration and purity was determined by NanoDrop (Thermo Scientific). Cells growth kinetics was monitored by the measurement of optical density at 600 nm. Protein expression was induced by IPTG to a final concentration of 1 mM. Protein presence was confirmed by SDS-PAGE (8% resolving gel and 4% stacking gel).

### Fluorimetric NMN assay

Bacterial cells were separated from growth media by centrifugation at 2200 g. The supernatant was collected separately and the cells were re-suspended in the same volume of water and lysed by sonication using a sonicator cell disruptor model W185F (Heat Systems-Ultrasonic Inc) on ice in 3 cycles of 30 seconds with a 30 seconds pause. The fluorimetric derivatization method developed by Zang *et al*.^47^ was adapted for 96 well plates with a 250 µL final volume per well, consisting of 69 µL sample, 27.7 µL 20% acetophenone in DMSO and 27.7 µL 2 M KOH. After 2 minutes of incubation on ice, a volume of 125 µL 88% formic acid was added to each well and the plate was incubated at 37 °C for 10 min. The UV emission was measured at 445 nm on a TECAN GENios microplate reader at excitation wavelength of 382 nm. Calibration curve in the concentration range 0.0625 × 10^−2^–4 × 10^−2^ mM with coefficient of correlation 0.99 was obtained by interpolating 8 standard sample dilutions of an external standard NMN (Sigma N3501-25MG). Lysate of bacterial cells from uninduced culture was used as control. The productivity data were expressed as mg NMN per g of protein. The protein concentration was determined by Bradford method^48^ using bovine serum albumin as standard.

### Statistical methods

Variance analysis was performed using OpenOffice Calc software. Each data point was plotted as mean value ± standard deviation of at least 3 independent experiments with significance calculated with Student’s *t*-test. Statistical significance was defined as P ≤ 0.05.

## Electronic supplementary material

Supplementary figures
