# Supplementary material for: β-nicotinamide mononucleotide (NMN) production in Escherichia coli
Source: Sci Rep. 2018 Aug 16;8:12278. doi: 10.1038/s41598-018-30792-0 (PMC6095924; doi:10.1038/s41598-018-30792-0)
Supplement: Supplementary file 1 — Supplementary figures [file 41598_2018_30792_MOESM1_ESM.pdf]

β-nicotinamide mononucleotide (NMN) production in Escherichia coli

George Cătălin Marinescu [1,2*], Roua-Gabriela Popescu [1,2], Gheorghe Stoian[1], Anca Dinischiotu[1]

1 Department of Biochemistry and Molecular Biology, University of Bucharest, Bucharest 050095, Romania

2 Independent Research Association, Bucharest 012416, Romania

*Corresponding author. George Cătălin Marinescu, Independent Research Association, Bucharest 012416, Romania; Department of Biochemistry and Molecular Biology, University of Bucharest, Bucharest 050095, Romania; E-mail: Catalin.Marinescu@independent-research.ro

Supplementary Figures

Supplementary Fig. S1

```
sp|Q99KQ4|NAMPT_MOUSE       ----MNAAAEAEFNILLATDSYKVTHYKQYPPNTSKVYSYFECREKKTENSKVRKVKYEE
tr|G1U9V7|G1U9V7_HAEDC      MDNLLNYSSRASAIPSLLCDFYKTSHRIMYPECSQIIYSTFTPRSNE------QAPYLTQ
tr|Q8EFJ1|Q8EFJ1_SHEON      ----------MYLNPVTAIDGYKVDHRRQYPDNTQVIFSNLTARKS-------RRGYTDQ
                                               * **. *   **  :. ::* :  *..       :     :

sp|Q99KQ4|NAMPT_MOUSE       TVFYGLQYILNKYLKGKVVTKEKIQEAKEVYREHFQDDV-----FNERGWNYIL-EKYDG
tr|G1U9V7|G1U9V7_HAEDC      VVSFGFQAFIIKYLIHYFNDNFFSR-DKHDVVTEYSAFIEKTLQLEDTG-EHIAKLHELG
tr|Q8EFJ1|Q8EFJ1_SHEON      MVFFGLQYFIKHYLIDSWNRDFFQQ-PKEQVICQFSRRINNYLGPNNVGTQHIEELHDLG
                             * :*:* :: :**      .   :  *.   .:.   :      :: * ::*   :  *

sp|Q99KQ4|NAMPT_MOUSE       HLPIEVKAVPEGSVIPRGNVLFTVENTDPECYWLTNWIETILV-QSWYPITVATNSREQK
tr|G1U9V7|G1U9V7_HAEDC      YLPIRIKAIPEGKTVAIKVPVMTIENTHSDFFWLTNYLETLINVSLWQPMTSASIAFAYR
tr|Q8EFJ1|Q8EFJ1_SHEON      YLPIKIMALPEGSVYPLKVPCLILYNTDERFFWLTNYLETILSANVWGMCTSATTALQYR
                            :***.: *:***..       : : **.   :****::**::  . *   * *: :   :

sp|Q99KQ4|NAMPT_MOUSE       KILAKYLLETSGNLDGLEYKLHDFGYRGVSSQETAGIGASAHLVNFKGTDTVAGIALIKK
tr|G1U9V7|G1U9V7_HAEDC      TALIKFANETCDNQEHVPFQSHDFSMRGMSSLESAETSGAGHLTSFLGTDTIPALSFVEA
tr|Q8EFJ1|Q8EFJ1_SHEON      KIFEAYALETDGDIAFVDWQGHDFSFRGMYGVEAAIMSGAAHLLSFTGTDTIPAIDFLEQ
                            . :  :  **  :   : :: ***. **: . *:*  ..:.** .* ****: .: :::

sp|Q99KQ4|NAMPT_MOUSE       YYGTK--DPVPGYSVPAAEHSTITAWGKDHEKDAFEHI-VTQFSSVPVSVVSDSYDIYNA
tr|G1U9V7|G1U9V7_HAEDC      YYGS---SSLIGTSIPASEHSVMSSHGVD-ELSTFRYL-MAKFPHNMLSIVSDTTDFWHN
tr|Q8EFJ1|Q8EFJ1_SHEON      YYLADSDKELVGGSVPATEHSVMCAGGMENELETFRRLIEDIYPTGIVSIVSDSWDFWQV
                            ** :   . : * *:**:***.: : * : * .:*. :    :    :*:***: *::.

sp|Q99KQ4|NAMPT_MOUSE       CEKIWGEDLRHLIVSRSTEAPLIIRPDSGNPLDTVLK-------------VLDILGKKFP
tr|G1U9V7|G1U9V7_HAEDC      ITV-NLPLLKQEIIARPENARLVIRPDSGNFFAIICGDPTADT-EHERKGLIECLWDIFG
tr|Q8EFJ1|Q8EFJ1_SHEON      MTE-FTLALKDRILAR--DGKVVFRPDTGCPVKIICGDPQAPIGSPEYKGAIECLWDVFG
                                    *:. *::*  :. :::***:*  .  :                :: * . *

sp|Q99KQ4|NAMPT_MOUSE       VTENSKGYKLLPPYLRVIQGDGVDINTLQEIVEGMKQKKWSIENVSFGSGGALLQKLTRD
tr|G1U9V7|G1U9V7_HAEDC      GTVNQKGYKVINPHIGAIYGDGVTYEKMFKILEGLQAKGFASSNIVFGVGAQTYQRNTRD
tr|Q8EFJ1|Q8EFJ1_SHEON      GSTTAKGYKLLDSHVGLIYGDSITIERAEAICAGLKAKGFASTNIVFGIGSFTYQHVTRD
                             : . ****::  ::  * **.:  :    *  *:: * ::  *: ** *.   *: ***

sp|Q99KQ4|NAMPT_MOUSE       LLNCSFKCSYVVTNGLGVNVFKDPVADP-NKRSKKGRLSLHRTPAGNFVTLEEGKGDLEE
tr|G1U9V7|G1U9V7_HAEDC      TLGFALKATSITINGEEKAIFKNPKTDDGFKKSQKGRVKVLSRDT---YV--DGLTSADD
tr|Q8EFJ1|Q8EFJ1_SHEON      TDGYAVKATFAKVDGKDREIFKDPKTDDGTKKSAKGLVAVFKDEQGQFYL--KDQASWQD
                                :.*.:    :*    :**:* :*   *:* ** : :            .   . ::

sp|Q99KQ4|NAMPT_MOUSE       YGHDLLHTVFKNGKVTKSYSFDEVRKNAQLNIEQDVAPH
tr|G1U9V7|G1U9V7_HAEDC      FSDDLLELLFEDGKLLRQTDFDEIRQNLLVSRTTL----
tr|Q8EFJ1|Q8EFJ1_SHEON      VNNCEFVPVFADGELLTEYSLADIRARLAASRR------
                              .  :  :* :*::  . .: ::* .   .
```

Supplementary Fig. S1. Amino acid sequence comparison of nicotinamide phosphoribosyl transferase (Nampt) from *Mus musculus* (sp|Q99KQ4|NAMPT_MOUSE, accession no. NP_067499.2 [UniProt]), putative NadV from *Haemophilus ducreyi* (tr|G1U9V7|G1U9V7_HAEDC accession no. NP_957670.1 [UniProt]) and NadV from *Shewanella oneidensis* MR-1 (tr|Q8EFJ1|Q8EFJ1_SHEON, accession no. NP_717588.1 [UniProt]). "*" indicates positions which single, fully conserved residue, ":" indicates conservation between groups of strongly similar properties - scoring > 0.5 in the Gonnet PAM 250 matrix and "." indicates conservation between groups of weakly similar properties - scoring =< 0.5 in the Gonnet PAM 250 matrix. The alignment was performed using the Clustal software (http://www.clustal.org/omega/).

Supplementary Fig. S2

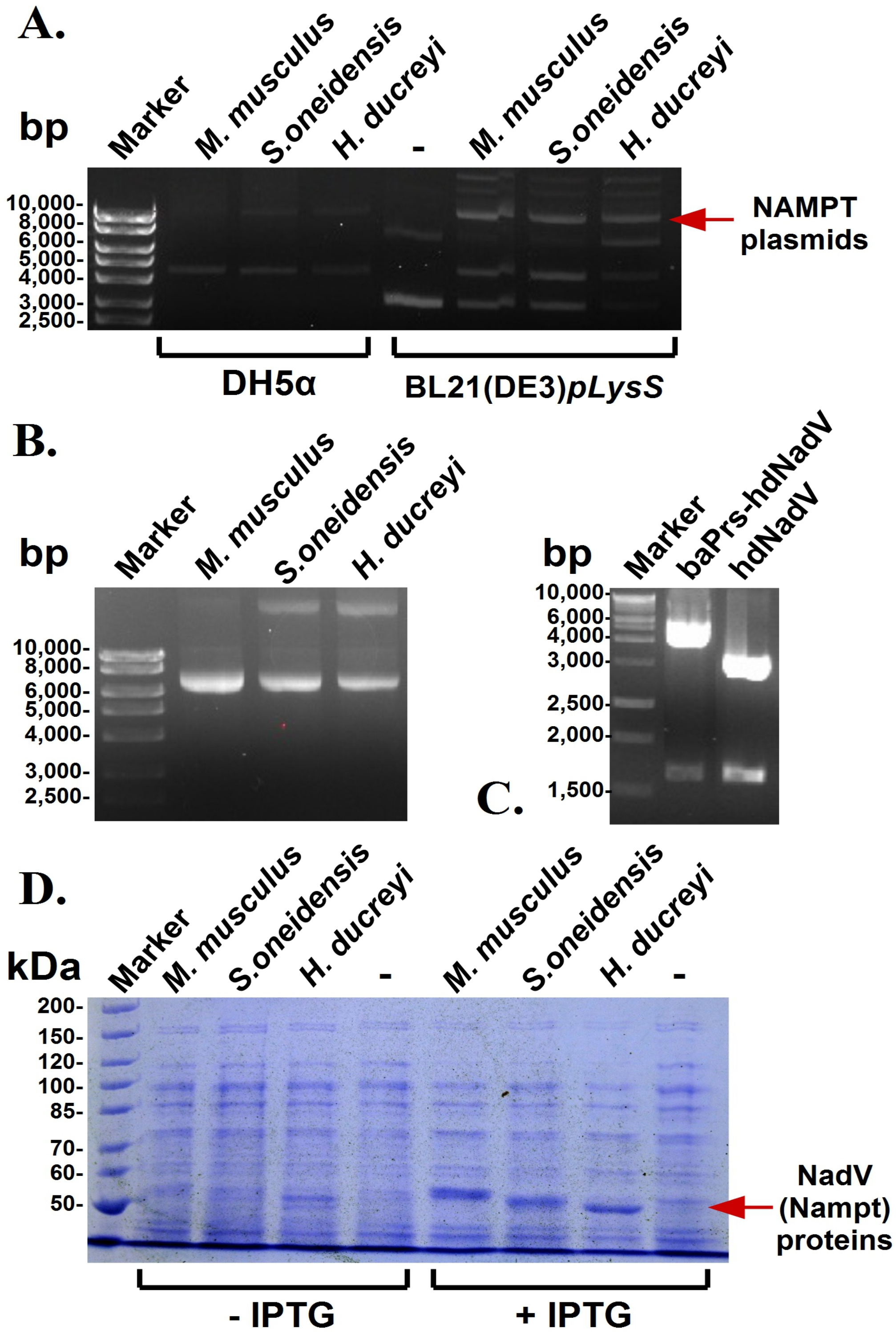

Supplementary Fig. S2.

A. Agarose gel electrophoresis of circular plasmid DNA isolated from *Escherichia coli* DH5α transformed culture with nadV (NAMPT) gene (lane 2, 3, 4); from untransformed *E. coli* BL21(DE3)pLysS culture (lane 5) and transformed with nadV (NAMPT) gene (lane 6, 7, 8) in the pET28a(+) vector; first lane - molecular weight marker (BIOLINE HyperLadder™ 1kb).

B. Agarose gel electrophoresis of plasmidial DNA from *Escherichia coli* DH5α (Addgene #25630);

and for pET-28a(+) backbone transformed with nadV genes (GenScript synthesis); first lane - molecular weight marker (BIOLINE HyperLadder™ 1kb).

C. Agarose gel of PCR amplification products (using T7 promoter and terminator primers) from *Escherichia coli* DH5α transformed culture with bicistronic respective nadV vector; first lane - molecular weight marker (1 Kb DNA Ladder, Promega).

D. SDS-PAGE analysis of NadV (Nampt) recombinant protein expression from *Escherichia coli* BL21(DE3)pLysS transformed with nadV (NAMPT) genes (lane 2, 3, 4) and supplemented with IPTG (lane 6, 7, 8); untransformed control (lane 5). Lane 1 - molecular weight marker (Thermo Scientific™ PageRuler™ Unstained Protein Ladder) (10-200 kDa).

Supplementary Fig. S3

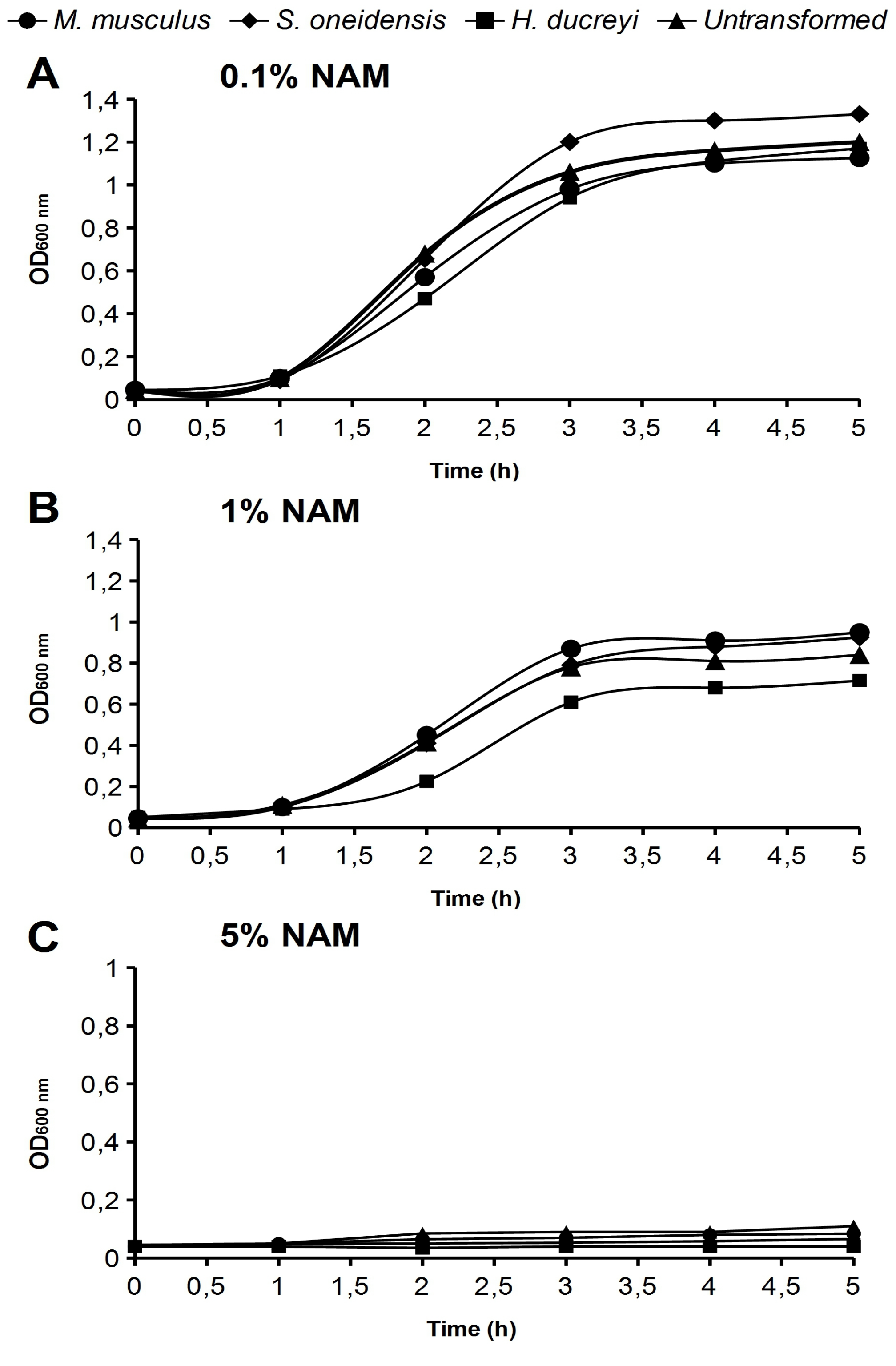

Supplementary Fig. S3. The growth (OD600) curve for transformed *Escherichia coli* BL21(DE3)pLysS transformed with pET-28a(+) nadV (NAMPT) gene carrying vector in growth medium supplemented with 0.1% (A), 1% (B) and 5% NAM (C).

Supplementary Fig. S4

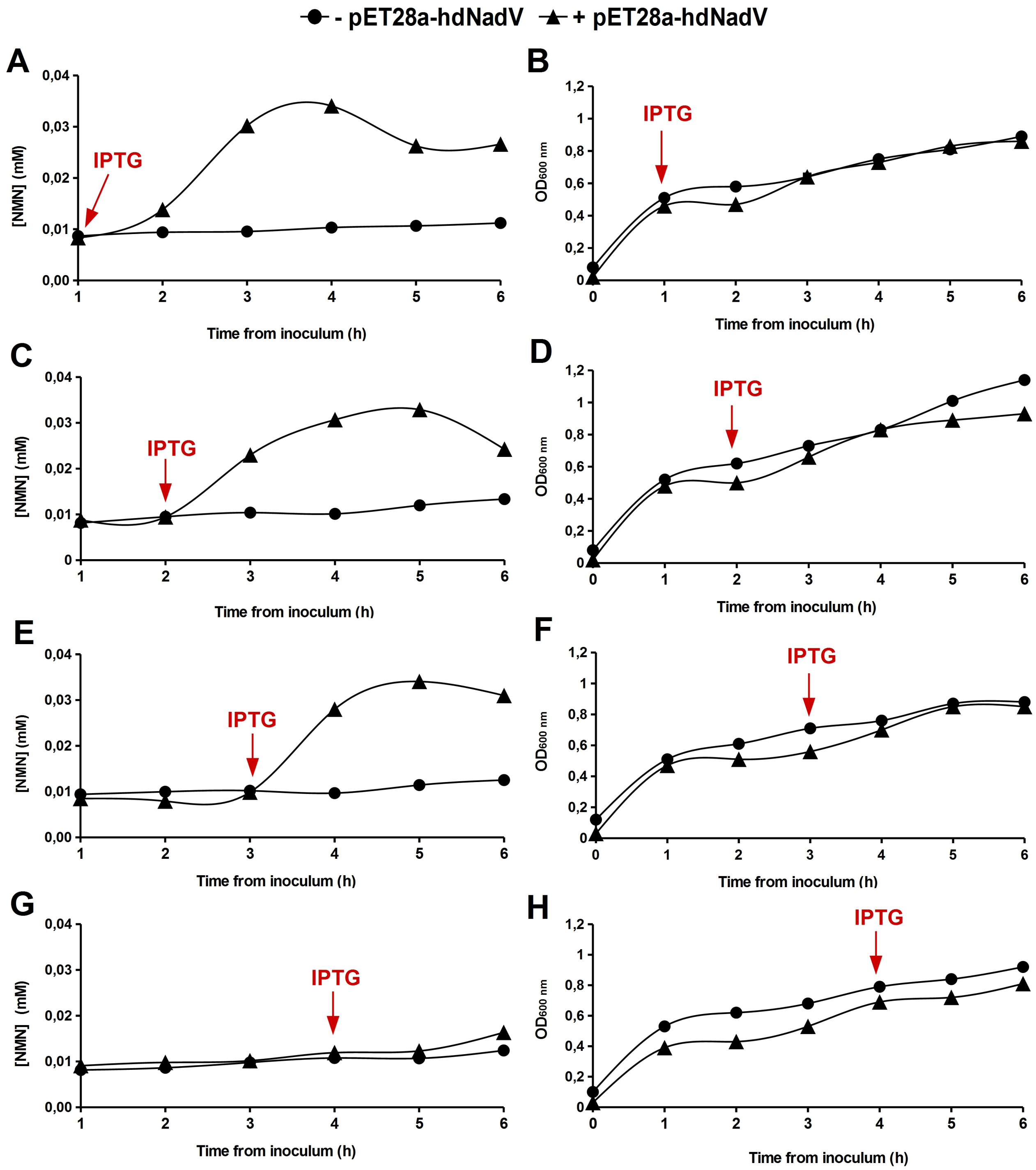

Supplementary Fig. S4. NMN concentration (A, C, E, G) and culture density (B, D, F, H) determined hourly in *Escherichia coli* BL21(DE3)pLysS transformed with nadV gene from *Haemophilus ducreyi* (pET28a-hdNadV) shake flasks culture, nadV expression was induced by addition of IPTG to a final concentration of 1 mM after one hour (OD600= 0.46) (A, B), two hours (OD600= 0.5) (C, D), three hours (OD600= 0.56) (E, F) and respective four hours (OD600= 0.69) (G, H) after inoculum.
